# Establishing integrated chronic non-communicable disease management clinics to address China’s looming health burden

**DOI:** 10.1186/s40779-025-00616-0

**Published:** 2025-05-30

**Authors:** Wen-Jun Tu, Xia Zhang, Hong-Qi Wang, Yang-Yi Fan, Jian-Lei Cao, Yan-Long Ren, Shi Bu, He-Tao Bian, Wei Yue, Ji-Lai Li, Long-De Wang

**Affiliations:** 1 Department of Neurosurgery, Beijing Tiantan Hospital, Capital Medical University, Beijing, 100070 China; 2 Translational Laboratory of Clinical Medicine, Chinese Institute for Brain Research & Beijing Tianan Hospital, Beijing, 100206 China; 3 Department of Neurology, Aerospace Center Hospital, Peking University Aerospace School of Clinical Medicine, Beijing, 100049 China; 4 Department of Anatomy, School of Basic Medical Sciences, Capital Medical University, Beijing, 100069 China; 5 Department of Neurology, Peking University People’s Hospital, Beijing, 100044 China; 6 Department of Cardiology, Wuhan University Zhongnan Hospital, Wuhan, 430071 China; 7 Department of Cardiology, Beijing Anzhen Hospital, Capital Medical University, Beijing, 100029 China; 8 Department of Endocrinology, China-Japan Friendship Hospital, Beijing, 100029 China; 9 Department of Stroke Prevention and Transformation, Xuanwu Hospital Capital Medical University, Beijing, 100053 China; 10 Department of Neurology, Tianjin Huanhu Hospital, Tianjin, 300350 China; 11 School of Public Health, Peking University, Beijing, 100191 China

**Keywords:** Chronic non-communicable disease (NCD), Clinic, Public health, China

Dear Editor,

The National Health Commission (NHC) of China has championed the creation of weight management clinics to confront the growing crisis of overweight and obesity. Forecasts indicate that by 2030, the prevalence of these conditions among Chinese adults and children could climb to 70.5% and 31.8%, respectively [1]. This troubling trajectory, alongside an increasing burden of chronic illnesses such as diabetes, hypertension, and cardiovascular diseases, underscores the need for immediate action. Instead of focusing solely on weight management clinics, we propose that medical institutions prioritize the establishment of comprehensive chronic disease management clinics, building upon the foundation of weight management clinics to holistically address obesity, aging, and healthcare system challenges.

China’s obesity rates have surged, making it one of the nations with the largest obese populations in the world [1]. According to the China Nutrition and Chronic Disease Report (2020), the prevalence of overweight and obesity among Chinese residents has been on the rise [2]. Strikingly, more than half of adult residents fall into the overweight or obese category, and the rates of overweight and obesity among children and adolescents aged 6–17 has reached 19.0%, while the rate for those under 6 years old is 10.4% [2]. Far from being an isolated issue, obesity serves as a critical risk factor for chronic diseases (Additional file 1: Fig. S1). At the same time, China’s aging population is expanding rapidly, with 267 million individuals aged 60 and older by 2021, comprising 18.9% of the total population [3]. This aging demographic, coupled with the rising prevalence of chronic conditions, is expected to propel obesity-related healthcare costs to 418 billion RMB by 2030, equivalent to 21.5% of national medical spending [4]. These staggering statistics highlight the limitations of relying solely on standalone weight clinics.

The NHC’s Obesity Diagnosis and Treatment Guidelines (2024) endorse a multidisciplinary team (MDT) approach to standardize obesity care. However, obesity represents just one dimension of a wider chronic disease spectrum. Older adults frequently contend with multiple comorbidities, such as cardiovascular diseases, diabetes, and osteoarthritis, necessitating cohesive rather than fragmented interventions. Integrated chronic non-communicable disease (NCD) management clinics, blending expertise from endocrinology, cardiology, and nutrition, could provide personalized care, slow disease progression, and enhance resource efficiency [5].

Global evidence reinforces the value of this strategy. For example, chronic care programs in the United States have successfully lowered hospitalization rates and costs through coordinated care [6]. China could adapt this model by building on its “Weight Management Year” initiative, weaving obesity prevention into a broader chronic disease framework to alleviate the healthcare strains of an aging society. A promising step in this direction is China’s ongoing promotion and establishment of “stroke clinics”, which focus on stroke patients and high-risk groups, striving for comprehensive chronic disease management [7]. Stroke clinics can provide effective full-process management for individuals at high risk of stroke (e.g., controlling risk factors such as hypertension, diabetes, dyslipidemia, lack of exercise, smoking, and alcohol consumption) as well as stroke patients (addressing rehabilitation needs and preventing stroke recurrence) [8] (Additional file 1: Fig. S2). A study from Pakistan indicates that the creation of structured stroke clinics, paired with an algorithmic approach to stroke management in primary care, could substantially enhance the adoption of evidence-based secondary prevention strategies in developing countries [9]. A retrospective cohort study involving 16,468 consecutive patients with ischemic stroke or transient ischemic attack demonstrates that referral to a stroke prevention clinic is linked to a 25% reduction in mortality following such events [10]. This finding bolsters the case that outpatient stroke units may be as effective as their inpatient counterparts. Prior evidence has consistently shown that stroke-unit care correlates with a decreased probability of death or disability [10].

To strengthen the feasibility of establishing integrated NCD management clinics, we proposed an implementation framework. These clinics should be staffed by an MDT comprising general practitioners, endocrinologists, cardiologists, neurologists, dietitians, rehabilitation specialists, and nurse coordinators to deliver holistic and continuous care. To optimize infrastructure and patient access, clinics could be co-located within existing community health centers or tertiary hospitals. Standardized referral pathways, supported by digital health tools such as interoperable electronic medical records and remote monitoring platforms, should be established to ensure seamless transitions across care levels. Initial implementation could be piloted in provinces with high NCD burdens, with key performance indicators including reduced hospitalizations, improved chronic disease control metrics, and enhanced patient satisfaction. Aligning this initiative with national frameworks, such as the Healthy China 2030 strategy and Family Doctor Contract Services, will be pivotal for scalability and long-term sustainability.

We urge healthcare institutions to accelerate the development of integrated chronic disease management clinics and encourage the international community to take notice. The converging challenges of obesity and aging require systemic, cohesive solutions rather than disjointed efforts. Looking forward, these clinics could become a cornerstone of China’s Healthy China 2030 strategy. By harnessing digital health innovations, such as remote monitoring and artificial intelligence (AI)-driven diagnostics, China can boost efficiency and extend care to underserved regions. International collaboration is equally critical; sharing data and insights with global health organizations could sharpen this approach. We hope the *Military Medical Research* will lead the charge in advocating for this movement, spurring cross-national innovation in aging and chronic disease prevention, and establishing China’s model as a global benchmark for public health excellence.

## Supplementary Information

**Additional file 1. Fig. S1** Overweight & obesity are one of the risk factors for chronic diseases. **Fig. S2** Stroke clinic with whole-course management.

## Abbreviations

NHC: National Health Commission
MDT: Multidisciplinary team
NCD: Chronic non-communicable disease
