# Supplementary material for: Establishing integrated chronic non-communicable disease management clinics to address China’s looming health burden
Source: Mil Med Res. 2025 May 30;12:25. doi: 10.1186/s40779-025-00616-0 (PMC12123705; doi:10.1186/s40779-025-00616-0)
Supplement: Supplementary file 1 — Additional file 1. Fig. S1 Overweight & obesity are one of the risk factors for chronic diseases. Fig. S2 Stroke clinic with whole-course management. [file 40779_2025_616_MOESM1_ESM.pdf]

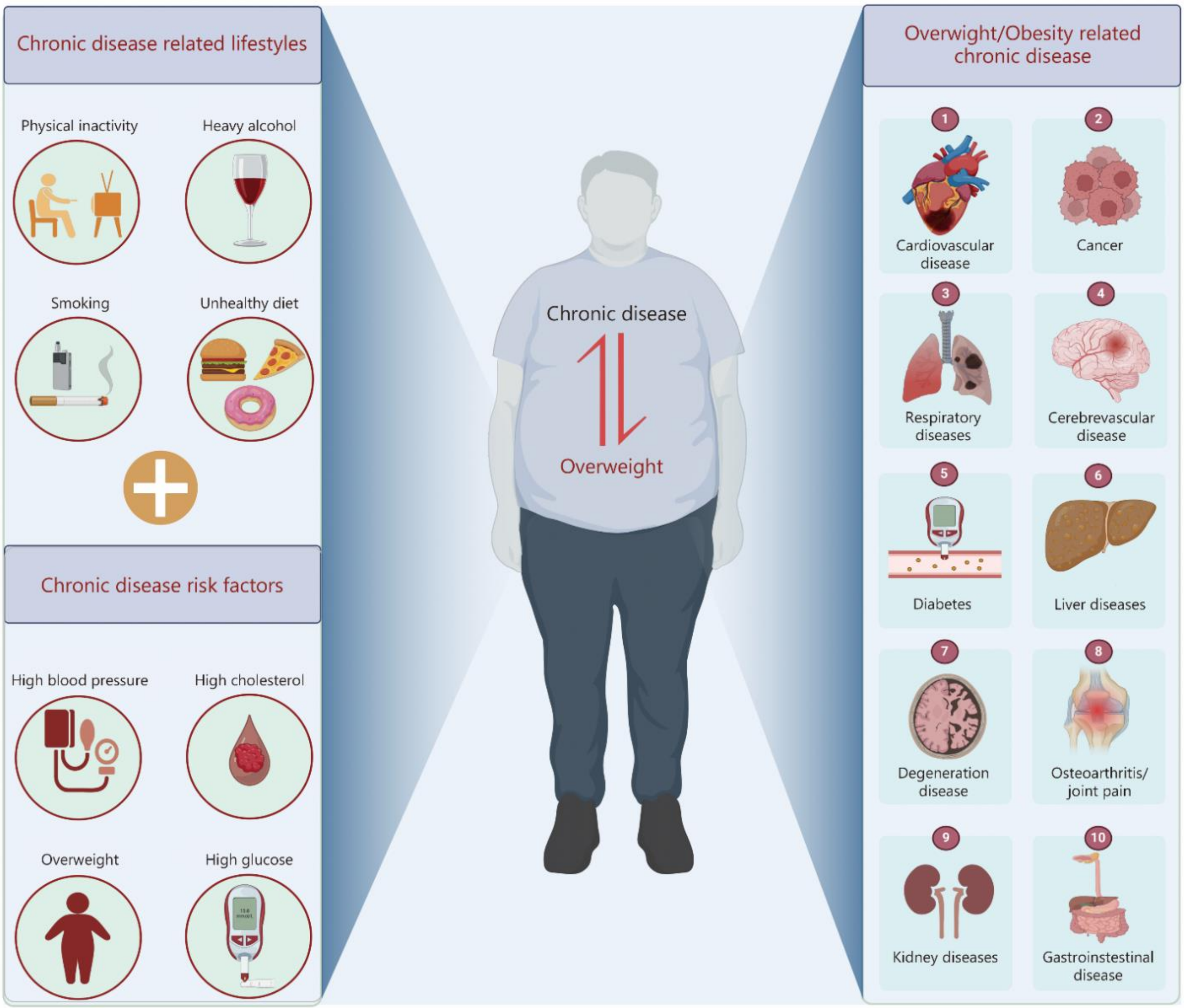

Fig. S1 Overweight & obesity is one of the risk factors for chronic diseases. It depicts the lifestyles and risk factors associated with chronic diseases, as well as the chronic diseases related to overweight and obesity.

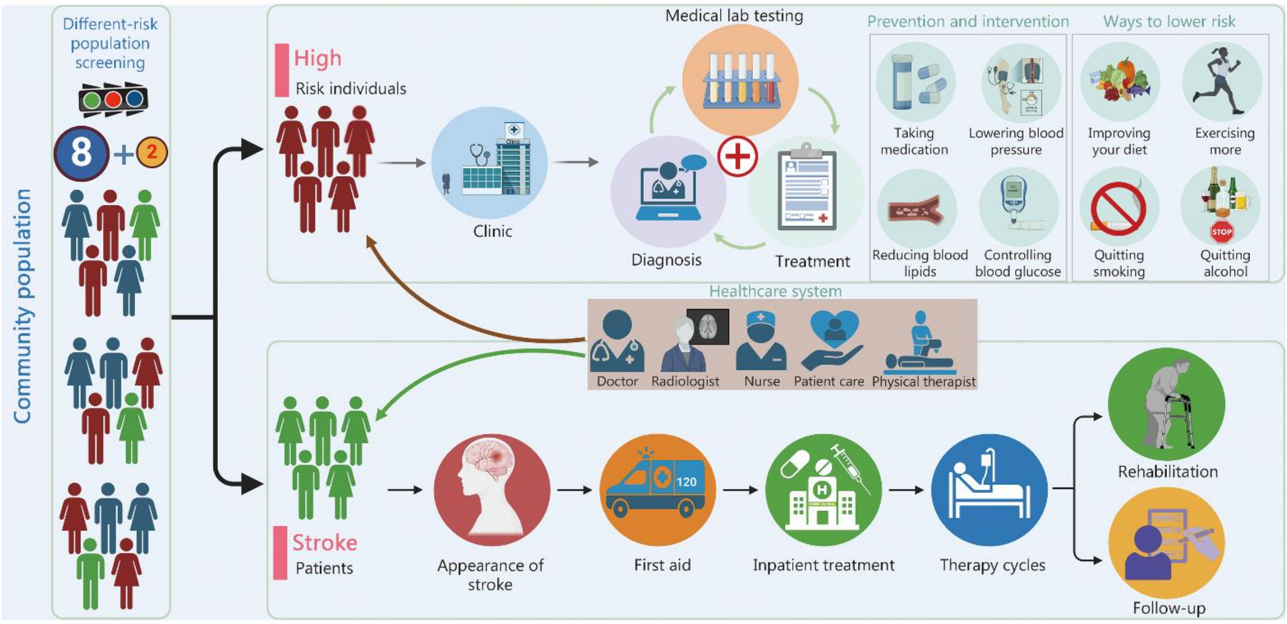

Fig. S2 Stroke clinic with whole-course management. It illustrates the stratified management principles for different risk levels after cerebrovascular disease screening in community populations: high-risk individuals are included in comprehensive stroke outpatient management; stroke patients are enrolled in an integrated stroke management model.
